# From Vineyard to Hydrogel: Antioxidant, Anti-Inflammatory, and Regenerative Potential of Grape Skin Extract in Diabetic Wound Repair

**DOI:** 10.3390/pharmaceutics17111464

**Published:** 2025-11-13

**Authors:** Jovana Bradić, Anica Petrovic, Jovana Joksimovic Jovic, Marko Simic, Vesna Stankovic, Sanja Matic, Marko Antonijević, Edina Avdovic, Vladimir Jakovljevic, Aleksandar Kocovic

**Affiliations:** 1 Department of Pharmacy, Faculty of Medical Sciences, University of Kragujevac, Svetozara Markovica, 69, 34000 Kragujevac, Serbia; 2 Center of Excellence for Redox Balance Research in Cardiovascular and Metabolic Disorders, Zmaj Jovina 30, 34000 Kragujevac, Serbiadrvladakgbg@yahoo.com (V.J.); 3 Department of Physiology, Faculty of Medical Sciences, University of Kragujevac, Svetozara Markovica, 69, 34000 Kragujevac, Serbia; 4 Department of Pathology, Faculty of Medical Sciences, University of Kragujevac, Svetozara Markovica, 69, 34000 Kragujevac, Serbia; 5 Institute for Information Technologies, University of Kragujevac, Jovana Cvijica bb, 34000 Kragujevac, Serbia; mantonijevic@uni.kg.ac.rs (M.A.);; 6 Department of Human Pathology, Sechenov First Moscow State Medical University, 8 Trubetskaya Street St., 119991 Moscow, Russia

**Keywords:** grape skin extract, bioactive hydrogels, diabetic wounds, inflammation, oxidative stress

## Abstract

**Background/Objectives:** This research aims to offer significant insights into the prospective application of bioactive hydrogels composed of alginate, gelatin, and grape skin extract from Serbia (GSE) for treating diabetic wounds, supporting the circular economy and environmental protection. **Methods:** An acute dermal irritation study was conducted according to OECD guidelines, revealing no visible signs of erythema or edema, confirming the hydrogel’s dermal safety. Afterwards, male Wistar rats were divided into four groups: untreated control (NC), silver sulfadiazine-treated (PC), hydrogel without extract (HG), and hydrogel with GSE (HG + GSE). Wound healing was assessed through a comprehensive approach that included macroscopic wound contraction; biochemical assessment of hydroxyproline content and oxidative stress markers (TBARS, SOD, CAT, GSH); quantification of inflammatory cytokines (TNF-α, IL-6); and histological examination of skin samples using hematoxylin–eosin (H&E) and Masson’s trichrome staining. **Results:** Daily HG+GSE application over 15 days accelerated wound closure, reaching 99.3% by day 15, surpassing PC (91.2%) and HG (87.7 ± 2.1%). Hydroxyproline levels followed a treatment-dependent pattern, with HG+GSE achieving the highest values throughout, reaching 6.78 ± 0.1 µg/mg dry tissue by day 15—more than double NC. The HG+GSE reduced lipid peroxidation while enhancing enzymatic and non-enzymatic antioxidant defenses and markedly lowered pro-inflammatory cytokine levels, indicating systemic anti-inflammatory activity. Histological analysis revealed faster re-epithelialization, increased collagen deposition, and more organized tissue architecture in the HG+GSE group. These outcomes are attributed to the sustained release of bioactive polyphenols such as naringin, caffeic acid, and epicatechin. **Conclusions:** Overall, this GSE-based hydrogel presents a multifunctional, biocompatible, sustainable, and effective strategy for diabetic wound care.

## 1. Introduction

Chronic wounds, particularly those associated with diabetes mellitus, present a significant clinical challenge due to impaired healing processes characterized by prolonged inflammation, reduced angiogenesis, and poor re-epithelialization. Diabetic foot ulcers are the most severe clinical manifestation of such impaired wound healing and the main reason for foot amputation [1]. As the global incidence of diabetes wounds continues to rise, the development of effective and biocompatible wound care strategies has become increasingly important [2]. Bioactive hydrogels (natural and synthetic), with their high water content and tissue-like physical properties, have emerged as promising wound dressing materials capable of maintaining a moist environment, delivering bioactive agents, and supporting tissue regeneration [3].

Although widely used, synthetic polymer hydrogels in wound care often face issues with skin compatibility, potential toxicity and lack of biodegradability [4]. This demonstrates the increasing demand for natural, bioactive hydrogel systems that actively aid in the healing process through their innate therapeutic qualities in addition to providing enhanced biocompatibility and sustainability. Because of their biodegradability, biocompatibility, and capacity to form stable matrices appropriate for biomedical applications, natural materials like gelatin and alginates are being extensively researched for the formulation of hydrogels [5]. The Alginate has been used to make hydrogels that enhance hemostasis and provide a supportive environment for cell migration and tissue regeneration during wound healing [6]. On the other hand, gelatin, a denatured form of collagen, mimics the extracellular matrix (ECM), providing essential cues for cell adhesion, proliferation, and differentiation [7]. Alginate and gelatin combine to form a synergistic matrix that fosters an optimal environment for wound healing by enhancing re-epithelialization, neovascularization, and ECM remodeling, processes commonly impaired in diabetic wounds [8].

In recent years, plant-derived bioactive compounds have gained attention as adjuncts in wound management strategies for their therapeutic potential and minimal side effects. Grape skin extract (GSE), considered as waste after wine production, contain polyphenols which can help neutralize reactive oxygen species (ROS), which are often elevated in diabetic wounds and contribute to delayed healing [9]. Grape skins from the Šumadija region of Serbia, renowned for their rich viticulture tradition, have been shown to possess strong anti-inflammatory and antioxidant properties [10,11]. These properties make GSE valuable components for the creation of novel therapeutic agent formulations for wound healing and other biomedical uses. They are also crucial for the prevention and management of conditions linked to oxidative damage. The incorporation of GSE into dietary supplements, bioactive hydrogels, or dermocosmetic products is a natural and efficient way to take advantage of their health benefits because of their high phenolic compound content and demonstrated biological activity. Given the increased interest in sustainable and natural sources of bioactive compounds, grape skins from the Serbian region stand out as a very promising raw material [10,11]. Building on our previous successful development and thorough characterization of eco-friendly alginate–gelatin hydrogels enriched with GSE, demonstrated to provide controlled release of polyphenols along with antioxidant and antimicrobial effects, we hypothesized that this novel formulation may effectively promote wound repair in rats [12]. We assumed that this formulation will specifically improve wound contraction, increase hydroxyproline content, and reduce oxidative stress and inflammation, exhibiting superior efficacy compared to standard silver sulfadiazine treatment.

Therefore, this study aimed to comprehensively assess the effectiveness of innovative GSE-loaded hydrogels in enhancing wound healing using a diabetic rat excision wound model through functional, biochemical, and histological analyses. Integrating GSE into biopolymers matrix not only introduces a sustainable, valorized byproduct of the wine industry, but also offers a natural and multifunctional alternative for developing advanced wound dressings with improved biocompatibility, antioxidant, and anti-inflammatory capacity. The results of this work may contribute to the advancement of phytochemical-loaded biomaterials for effective diabetic wound management.

## 2. Materials and Methods

### 2.1. Preparation of Grape Skin Extract (GSE)

GSE was procured through the method of ultrasound-assisted extraction (UAE), utilizing ethyl acetate as the solvent. Grape skins first underwent a washing process, followed by air drying, and were then ground to achieve a consistent particle size. In each extraction, 20 g of dried grape skin material were combined with 200 mL of ethyl acetate and subjected to sonication in an ultrasonic bath operating at 40 kHz and 150 W for a duration of 30 min at a temperature of 40 °C. After sonication, the mixtures underwent centrifugation at 4500 rpm for a duration of 10 min. The resulting supernatants were then collected and filtered using a 0.45 μm PTFE syringe filter. The filtrates obtained were concentrated at a reduced pressure of 40 °C with the aid of a rotary evaporator to eliminate any remaining solvent. Concentrated extracts were kept at 4 °C in a dark environment until they were needed for further applications. Extraction was conducted in triplicate to guarantee consistent results. More details about the extraction and tests of antioxidant, anti-inflammatory, and antimicrobial properties of GSE are present in the recently published manuscripts [10,11]. The extraction was performed using a solid-to-solvent ratio of 1:10 (*w*/*v*), corresponding to 20 g of dried grape skin material extracted with 200 mL of ethyl acetate under ultrasound (40 kHz, 150 W, 30 min, 40 °C). The final extract yield was 5.1% *(w*/*w*), as determined in our previous optimization study [10,11,13].

### 2.2. Preparation of Hydrogels Loaded with GSE (HG+GSE)

**Figure 1:**
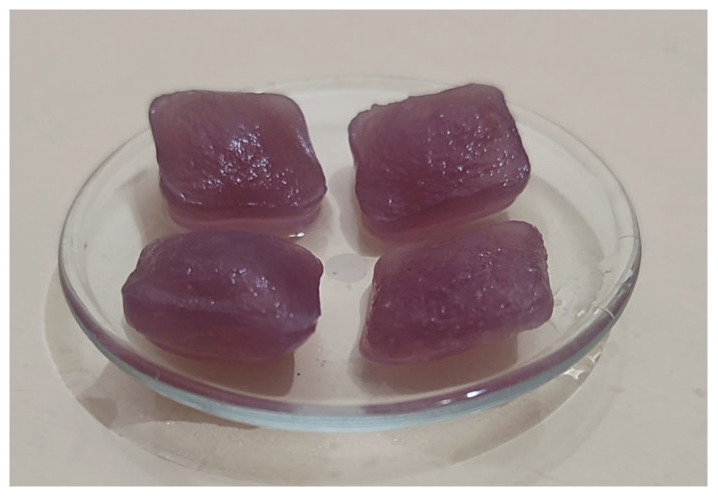
Photo of the HG+GSE.

Hydrogels loaded with GSE (HG+GSE) were prepared using a modified mold casting method. Grape skin extract (8 mg/mL) was dissolved in water, then alginate and gelatin (1:1 ratio, 3% each) were added and stirred at 500 rpm until uniform homogenization. The mixture was sonicated for 15 min to remove air bubbles and rested for 2 h at room temperature. It was then cast into 1 × 1 cm molds, matching the wound surface size, frozen at –20 °C for 2 h, and cross-linked by immersion in 0.5 M CaCl_2_ for 5 min. Finally, hydrogels were dried at room temperature before use (Figure 1). The hydrogel base (HG) was prepared in the same way, using water instead of the GSE solution [13,14]. All the chemicals used for extract and hydrogel preparation were purchased from Sigma-Aldrich, Darmstadt, Germany.

### 2.3. In Vivo Assessment of Safety and Wound Healing Potential of HG+GSE

#### 2.3.1. Ethics Statement

The study adhered fully to the European Directive 86/609/EEC on the protection of animals used for experimental and other scientific purposes, as well as the principles of Good Laboratory Practice (GLP). All experimental procedures were conducted at the Laboratory for pharmaceutical technology and the Center for preclinical and functional investigation, Faculty of Medical Sciences, University of Kragujevac, Serbia. The research protocol received prior approval from the Ethics Committee of the Faculty of Medical Sciences, University of Kragujevac (Approval No. 01-6856).

#### 2.3.2. Study Animals

Healthy adult male *Wistar albino* rats (200 ± 30 g body weight) were used in the in vivo experiments. The animals were obtained from the Military Medical Academy in Belgrade, Serbia. They were housed in clean cages under controlled conditions, including a 12-h light/dark cycle and a stable room temperature of 22 ± 2 °C. Food and water were available ad libitum throughout the study.

#### 2.3.3. Acute Dermal Irritation Assessment

The acute dermal irritation test was performed following the OECD Guideline 404 for testing of chemicals. A total of six male *Wistar albino* rats were used in this test. The fur on the dorsal region of each rat was carefully shaved, and the rats were randomly assigned to one of the two groups based on the applied formulation. The groups included:

1. BG group—rats treated with the base gel without GSE,
2. GSE group—rats treated with the gel with incorporated GSE (HG+GSE).

Half gram of the formulations was applied to defined shaved area of skin of each rat. Following application, the animals were housed in individual cages. Intensive monitoring was conducted during the first 4 h after application, after which the animals were monitored once daily for a period of 14 days. Parameters such as the presence and severity of erythema and edema were evaluated using the Draize scoring system. In this test a score of 0 indicates no observable reaction and a score of 4 indicates a severe erythema or edema [15]. 

#### 2.3.4. Induction of Diabetes Mellitus 

A single intraperitoneal injection of streptozotocin (dose of 50 mg/kg) was used to induce diabetes. Streptozotocin was dissolved in 1 mL of freshly prepared citrate buffer (0.05 M, pH 4.5). Prior to the injection, the rats starved for 12 h. After 72 h following streptozotocin administration, and after an additional 12 h fasting period, fasting blood glucose levels were measured using a portable glucometer (Accu-Check®, Basel, Switzerland). Rats with blood glucose levels that exceeded 11.1 mmol/L were classified as diabetic and included in the experimental protocol [16].

#### 2.3.5. Induction of Excision Wounds

Excision wounds were made one week following confirmation of diabetes in the animals. Anesthesia was achieved using an intraperitoneal injection of xylazine (10 mg/kg) and ketamine (5 mg/kg). Following anesthesia, the backs of animals were shaved and disinfected with 70% ethyl alcohol. After that, excision wounds measuring 1 × 1 cm were created using a scalpel and scissors [17]. Following the procedure, wound of each rat was photographed, and the rats were placed in individual cages.

#### 2.3.6. Treatment Protocol

Following excision wound induction, the rats were divided into four groups of 20 animals in each group based on their wound treatment. The groups were as follows:

1. Negative control (NC)—included rats with wounds that were left untreated;
2. Positive control (PC)—included rats with wounds treated with a cream containing 1% silver sulfadiazine;
3. Hydrogel base (HG)—included rats with wounds treated with a hydrogel without grape skin extract;
4. Hydrogel enriched with GSE (HG + GSE)—included rats with wounds treated with a hydrogel with incorporated grape skin extract.

All of the preparations were applied daily for 15 days. The hydrogels, prepared in molds to match the dimensions of the wounds, were placed on wounds using sterile pincers, while silver sulphadiazine cream was applied in the amount of 0.5 g per rat using sterile cotton swabs. The concentration of GSE was optimized in our previous formulation study, which confirmed its antioxidant and antimicrobial efficacy [13]. Wounds on each rat were photographed on days 3, 7, 11, and 15 using a digital camera for further analysis of the percentage of wound contraction. At these same time points, five animals from each group were first anesthetized using an intraperitoneal injection containing a mixture of ketamine (5 mg/kg) and xylazine (10 mg/kg) and then sacrificed by decapitation. Following sacrifice, the wound skin tissue was collected for subsequent biochemical and histological evaluations. All experimental rats were retained in the study, as none showed post-surgical mortality, notable morbidity, signs of infection, or abnormal behavior that would warrant exclusion. Every rat that completed the protocol was included in the final analysis for each parameter.

The primary outcome parameters evaluated in this study were wound contraction, hydroxyproline content measurement, assessment of oxidative stress and inflammatory markers in tissue samples and histological analysis. All chemicals used for diabetes induction, biochemical analyses of oxidative stress and hydroxyproline content, as well as for histological analyses, were purchased from Sigma-Aldrich (Darmstadt, Germany). Group allocation was conducted by one researcher, while treatment administration was performed by multiple researchers. Histological analysis was carried out by a pathology specialist. Outcome assessments and data analysis were independently performed by a researcher blinded to group assignments to ensure unbiased evaluation of the results.

No unanticipated adverse effects occurred during the study. Throughout the experiment, animals were carefully observed on a daily basis for any indicators of pain, distress, or unusual behavior, including alterations in movement, grooming, posture, and food or water consumption. If any unexpected adverse reactions or signs of suffering had been detected, appropriate measures such as additional pain relief or, if necessary, humane euthanasia would have been promptly implemented to uphold animal welfare. However, all animals responded well to the procedures, showing no significant signs of pain, infection, or treatment-related complications.

#### 2.3.7. Monitoring of Food and Water Consumption and Body Weight Changes in Rats

The animals were monitored for changes in body weight and consumption of food and liquids at four time points: days 3, 7, 11, and 15 post-treatment initiation.

#### 2.3.8. Wound Contraction Estimation

Wound areas were photographed immediately after injury and on days 3, 7, 11, and 15 post-wounding for wound contraction analysis. The wound area was then measured using graph paper and ImageJ software version 1.54 to calculate the extent of wound contraction. The percentage of wound contraction on each of these days was determined using the following formula [18]:

% Wound contraction = [(Initial wound area − Specific day wound area)/Initial wound area] × 100

### 2.4. Biochemical Parameters 

#### 2.4.1. Hydroxyproline Estimation

Dried wound tissues (12–18 h at 60 °C) were hydrolyzed in 6 M HCl (1:10 *w*/*v*) at 130 °C for 4 h, centrifuged (3000 rpm, 15 min), and supernatants reacted with chloramine-T and Ehrlich’s reagent. Absorbance at 557 nm was measured to quantify hydroxyproline (µg/mg dry tissue) using a standard curve [19].

#### 2.4.2. Estimation of Tissue Redox Parameters

Wound tissue samples were homogenized in cold phosphate-buffered saline (PBS) using a 1:10 weight-to-volume ratio. The homogenates were then centrifuged at 1200× *g* for 10 min at 4 °C. The obtained supernatants were collected into Eppendorf tubes and stored at −70 °C until the biochemical analyses were conducted. Key indicators of the antioxidant defense system such as reduced glutathione (GSH), superoxide dismutase (SOD), and catalase (CAT) as well as the pro-oxidative marker of lipid peroxidation measured via thiobarbituric acid (TBARS) were determined in the supernatant samples according to previously established protocols [20].

#### 2.4.3. Estimation of System Inflammatory Markers

The concentrations of IL-6 and TNF-α were measured using the sandwich ELISA method. Commercial Rat IL-6 (Elabscience, Wuhan, China, E-EL-R0015, Lot: GY0220221506) and TNF-α (Elabscience, Wuhan, China E-EL-R2856, Lot: GY59XH2P4751) ELISA kits were used to measure serum cytokines following the manufacturer’s protocol. The optical density (OD) was measured spectrophotometrically at a wavelength of 450 nm ± 2 nm. The OD value was proportional to the concentration of the corresponding cytokine. The concentrations of IL-6 and TNF-α in the samples were calculated by comparing the OD values with the standard curve. The intra- and inter-assay coefficients of variation for both cytokines were less than 10%.

### 2.5. Histological Analysis

Wound tissue samples were fixed in 10% buffered formalin at 4 °C for 24 h, dehydrated through graded isopropyl alcohol (70–100%), embedded in paraffin, and sectioned at 5 μm using a rotary microtome for histological analysis. After sectioning, tissue samples were stained with hematoxylin and eosin (H/E) to assess general tissue morphology and cellular structure. Additionally, Masson’s trichrome staining was performed to evaluate collagen deposition and extracellular matrix remodeling in the wound tissue [17,21]. Morphometric analysis of collagen content was performed using the software Image Pro-Plus software 7.0 (Media Cybernetics, Rockville, MD, USA), following previously described methodologies [22]. Results are expressed as percentages.

### 2.6. Statistical Analysis

Data was expressed as mean ± standard deviation. Before performing statistical analysis, normality of the data was assessed using the Shapiro–Wilk test. One-way analysis of variance (ANOVA) was performed for data with a normal distribution followed by Tukey’s post hoc test for multiple comparisons. All statistical evaluations were conducted using IBM SPSS statistics version 20 for Windows. A *p*-value of less than 0.05 was considered statistically significant, while *p*-value of less than 0.01 was considered highly statistically significant.

## 3. Results and Discussion

This study represents pioneering research focused on the in vivo evaluation of a hydrogel composed of natural biopolymers and GSE, a valuable byproduct of the wine industry, assessing both its safety and wound healing efficacy. Encouraged by our previous success in developing and characterizing alginate–gelatin hydrogel enriched with GSE that possesses antioxidant and antimicrobial activities [13], we extended this research to evaluate its in vivo wound healing potential. The hydrogel was formulated by combining alginate and gelatin to exploit their synergistic properties, since alginate offers a moist, biocompatible matrix conducive to healing, while gelatin supports cell adhesion and tissue regeneration [23,24]. The GSE used in this study, previously characterized in our published work [13], contains a diverse profile of phenolic acids and flavonoids, including high levels of epicatechin (5415 ± 39 mg × 10^−2^/g), naringin (3828 ± 54 mg × 10^−2^/g), caffeic acid (1051 ± 26 mg × 10^−2^/g), and other bioactive compounds such as gallic acid, rutin, myricetin, and quercetin. The detailed physicochemical characterization of the alginate–gelatin hydrogel, including FTIR and SEM analyses, was reported in our resent study [13], confirming the structural and intermolecular integrity of the Ca^2+^-crosslinked network. These material properties provided a solid basis for interpreting the hydrogel’s in vivo performance, as porosity, core–shell morphology, and hydrogen-bonding interactions likely influence bioactive release and tissue integration. Building upon these previously established characteristics, the present study focused on evaluating the in vivo wound-healing performance of the hydrogel system enriched with GSE in a diabetic model.

### 3.1. Skin Tolerability of HG+GSE in Rats

In the initial phase of the study, an acute dermal irritation test was conducted to evaluate the safety profile of the grape skin extract-enriched hydrogel (HG+GSE), which is a critical step in determining its potential to cause skin irritation [25]. This assessment was essential to ensure that the formulation would not induce any adverse cutaneous reactions upon application. No visible signs of dermal toxicity, such as erythema or edema, were observed during the 14-day observation period, indicating good tolerability of the hydrogel. Following the confirmation of its dermal safety, the study proceeded with the evaluation of HG+GSE’s wound healing efficacy in a rat excision wound model.

### 3.2. Therapeutic Effects of HG+GSE on Diabetic Wound Healing

**Figure 2:**
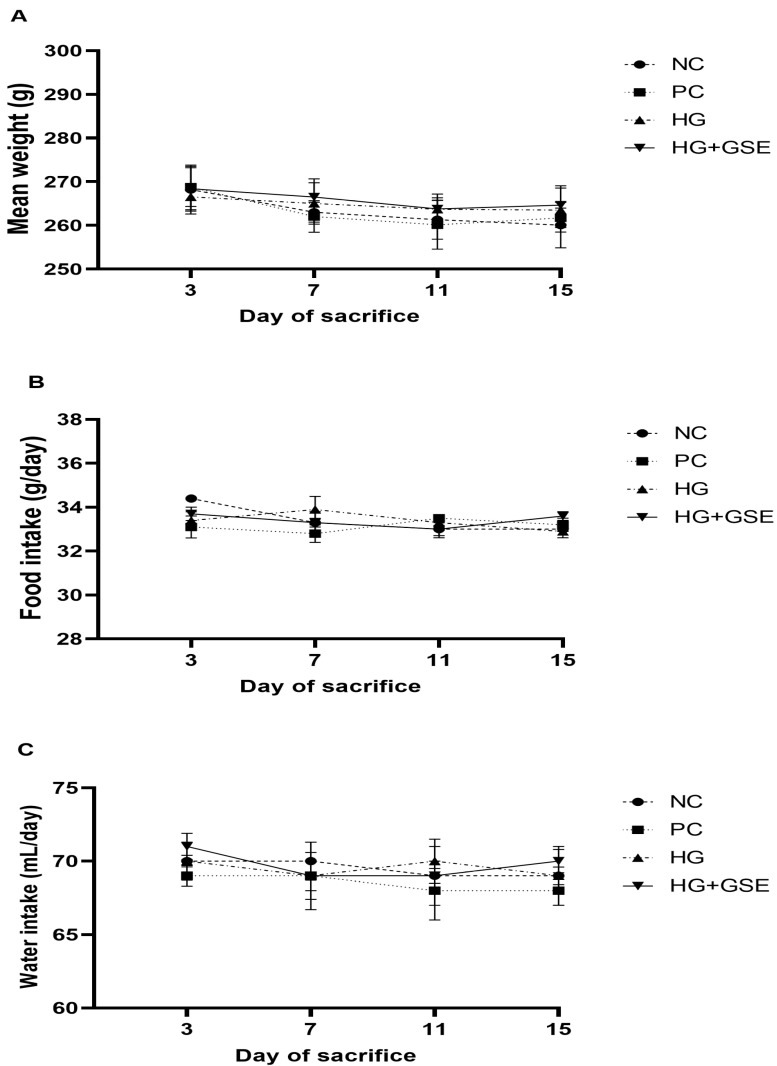
Body weight (**A**), food intake (**B**) and water consumption (**C**) changes in rats. Data represent mean ± SD. NC—nontreated wounds; PC—wounds treated with 1% silver sulfadiazine cream; HG—wounds treated with hydrogel (base hydrogel without extract) and HG+GSE—wounds treated with hydrogel with grape skin extract (GSE).

To evaluate the healing efficacy of the novel hydrogel, a full-thickness excision wound model in rats was employed, which effectively mimics acute clinical wounds and allows detailed observation of critical healing phases [26]. Firstly, food and water intake, along with body weight, were monitored on days 3, 7, 11, and 15 post-treatment to evaluate the metabolic status of diabetic rats and the possible systemic effects of the treatments. These parameters are important, as weight loss and changes in food and water intake can negatively impact wound healing. Slight variations in food and water consumption, along with body weight, were observed across all groups during the 15-day period, likely due to the combined effects of diabetes and wound injury. Although all groups displayed similar food and water intake trends, the HG+GSE group showed slightly higher food and water consumption (Figure 2A,B) and a modest increase in body weight (Figure 2C). Conversely, the untreated diabetic group (NC) experienced ongoing weight loss, reflecting the typical catabolic state in diabetes (Figure 2C). These outcomes demonstrate that HG+GSE may partially counteract systemic diabetic effects.

Moreover, healing efficacy of HG+GSE was demonstrated through functional measurements, biochemical analyses, and histological evaluation. 

#### 3.2.1. Wound Healing Progression Following HG+GSE Treatment

**Figure 3:**
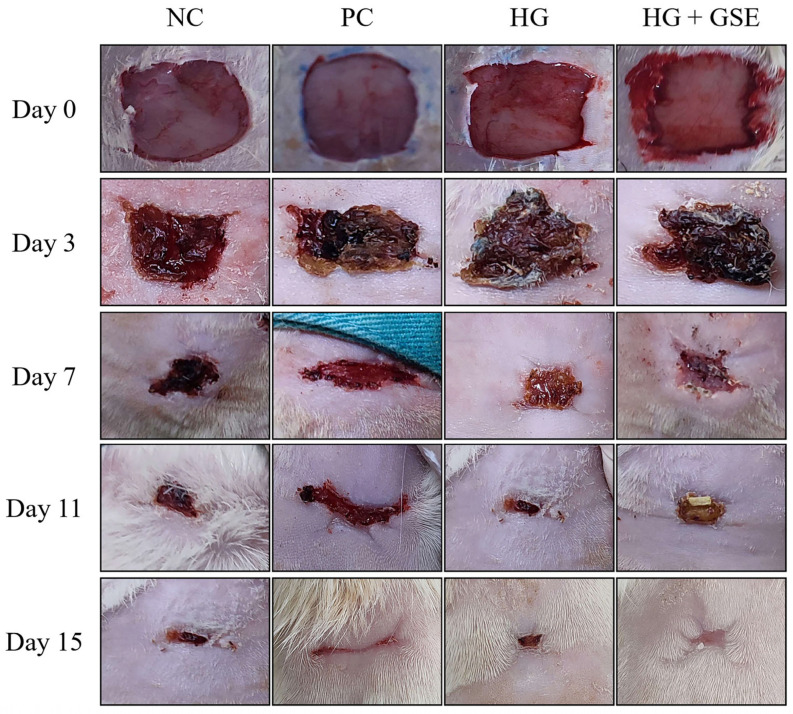
Wound healing progression in rats. NC—nontreated wounds; PC—wounds treated with 1% silver sulfadiazine cream; HG—wounds treated with hydrogel (base hydrogel without extract) and HG+GSE—wounds treated with hydrogel with grape skin extract (GSE).

**Figure 4:**
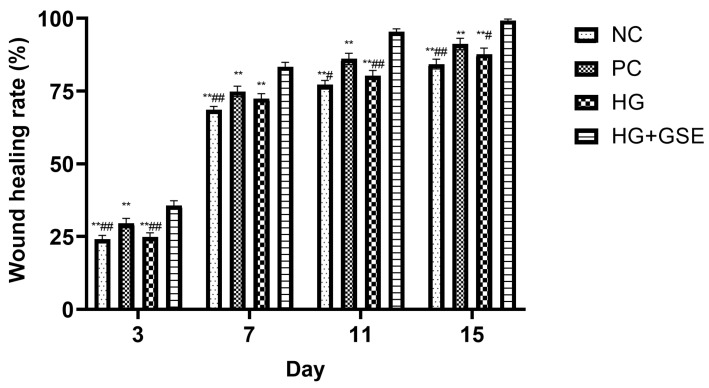
Rate of Wound Closure (%) During the Healing Process. Data represent mean ± SD. NC—nontreated wounds; PC—wounds treated with 1% silver sulfadiazine cream; HG—wounds treated with hydrogel (base hydrogel without extract) and HG+GSE—wounds treated with hydrogel with grape skin extract (GSE); ** statistically significant difference compared to HG+GSE at the level *p* < 0.01; ^#^ statistically significant difference compared to PC at the level *p* < 0.05; ^##^ statistically significant difference compared to PC at the level *p* < 0.01.

Wound healing progression was monitored on days 3, 7, 11, and 15 across four experimental groups: non-treated control (NC), silver sulfadiazine 1% cream (PC), hydrogel without extract (HG), and hydrogel containing grape skin extract (HG + GSE). Representative wound photographs at each time point are shown in Figure 3, while quantitative analysis of wound contraction is presented in Figure 4.

By day 3, the HG+GSE group already exhibited significantly enhanced healing (35.6 ± 1.7%) compared to the other groups, suggesting early modulation of inflammation and initiation of tissue regeneration. By day 7, wound closure in the HG+GSE group reached 83.4 ± 1.5%, significantly surpassing PC (74.9 ± 1.8%), HG (72.4 ± 1.7%), and NC (68.5 ± 1.2%). During this stage, the wound is actively undergoing proliferative phase and accelerated healing in the HG+GSE group is likely due to enhanced fibroblast migration and ECM deposition, supported by the extract’s antioxidative microenvironment [27]. Protective effects were sustained through days 11 and 15, with nearly complete healing observed in the HG+GSE group (95.4% and 99.3%, respectively), compared to 91.2% in PC group and 87.7 ± 2.1% in HG group by day 15, thereby demonstrating the hydrogel had capacity to support both the proliferative and remodeling phases of healing [27]. The HG-only group reached 87.7 ± 2.1% by day 15, indicating that while the hydrogel provided a moist healing environment, it lacked the bioactive stimulation provided by the extract.

These findings clearly show that the HG+GSE formulation significantly outperformed all other treatments in promoting wound contraction at each observed time point. The superior performance of the HG+GSE formulation is likely attributable to the combined benefits of a moist hydrogel matrix and controlled delivery of bioactive properties of grape skin polyphenols to the wound site. Our previous phytochemical analysis identified naringin, epicatechin, and caffeic acid as dominant constituents of the observed extract, with epicatechin and naringin being particularly abundant [13]. The superior wound contraction observed in the HG+GSE group can be mechanistically linked to naringin’s ability to enhance fibroblast migration and proliferation through MMP-2 and MMP-9 activation, which facilitate the breakdown of provisional matrix and enable cellular movement into the wound bed [28,29,30]. Literature evidence strongly supports the role of naringin in enhancing wound contraction and reducing local inflammation [28]. Furthermore, biomaterial-based studies have demonstrated that naringin promotes vascularization and antioxidant activity, facilitating faster and more effective healing [31]. The combined antioxidant effects of epicatechin and caffeic acid likely contributed to reducing oxidative stress-mediated cellular damage, thereby allowing normal progression through the healing phases. Additionally, caffeic acid’s dual action in suppressing pro-inflammatory cytokines while activating antioxidant defenses through the Nrf2/HO-1 pathway creates an optimal environment for tissue regeneration [32,33,34]. The wound healing effects exhibited by HG+GSE hydrogel are likely driven in part by the well-documented bioactive properties of caffeic acid reported in earlier studies [35,36]. Therefore, the therapeutic benefits of the HG+GSE formulation can be reasonably attributed to the synergistic action of the high naringin, epicatechin, and caffeic acid content gradually delivered via the hydrogel scaffold, with each compound addressing different aspects of the impaired healing cascade characteristic of diabetic wounds. Our previous study [13] confirmed that the sustained release of these polyphenols from the hydrogel occurs through anomalous (non-Fickian) transport, suggesting that release is governed by both Fickian diffusion and polymer relaxation/degradation. 

#### 3.2.2. Molecular Indicators of Healing: Collagen Synthesis, Oxidative Balance, and Inflammation 

**Figure 5:**
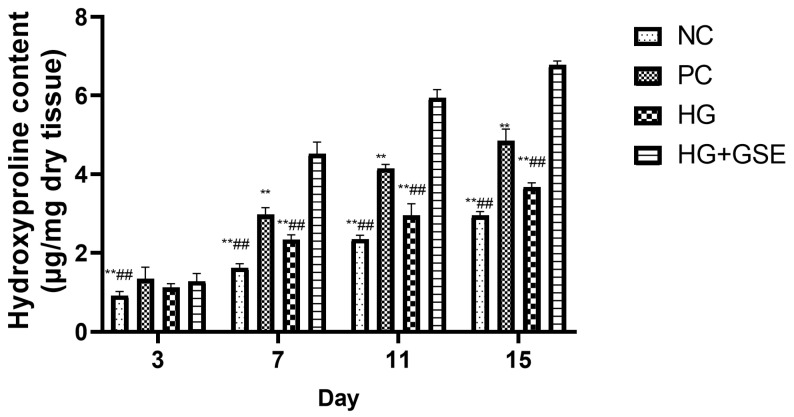
Hydroxyproline Content in Wound Tissue Following Treatment. Data represent mean ± SD of three independent experiments. NC—nontreated wounds; PC—wounds treated with 1% silver sulfadiazine cream; HG—wounds treated with hydrogel (base hydrogel without extract) and HG+GSE—wounds treated with hydrogel with grape skin extract (GSE); ** statistically significant difference compared to HG+GSE at the level *p* < 0.01; ^##^ statistically significant difference compared to PC at the level *p* < 0.01.

Hydroxyproline is commonly used as a dependable marker for assessing collagen accumulation and overall protein levels at the wound site, reflecting the advancement of the healing process into the remodeling phase. Quantifying hydroxyproline levels provides insight into new collagen synthesis and matrix maturation, both essential for restoring tissue strength and integrity during the later stages of repair [37,38]. Accordingly, hydroxyproline content in skin tissue was measured on days 3, 7, 11, and 15 to evaluate collagen deposition and tissue remodeling throughout the healing process (Figure 5).

A clear treatment-dependent pattern emerged, with the HG+GSE consistently showing the highest hydroxyproline levels at all time points. Namely, on day 3, the HG+GSE group already showed a modest but noticeable increase compared to NC and HG. Additionally, PC reached 1.34 ± 0.3 µg/mg dry tissue, slightly higher than HG+GSE, though not statistically significant at this early stage. These values suggest the onset of collagen synthesis had begun in all treated groups but was still in the early phase of deposition. Additionally, by day 7, the differences among groups became even more pronounced. The HG+GSE group exhibited a hydroxyproline level which was approximately 2.8 times higher than NC and nearly 2 times higher than HG. Even compared to the PC group, HG+GSE showed a significantly enhanced effect, indicating accelerated extracellular matrix formation likely driven by the antioxidant and regenerative activity of grape skin polyphenols. Moreover, on day 11, the hydroxyproline level in HG+GSE group increased further, remaining significantly higher than PC, HG and NC groups. At this point, hydroxyproline level in the HG+GSE group was more than 2.5 times higher than NC group and approximately double that of HG group, demonstrating robust matrix maturation and sustained fibroblast activity. At the end of the treatment period (day 15), the HG+GSE group achieved the highest hydroxyproline content (6.78 ± 0.1 µg/mg dry tissue), clearly surpassing PC, HG and NC groups. Notably, the HG+GSE value was more than double that of the NC group, highlighting its capacity to support collagen-rich tissue remodeling well into the later stages of wound healing. These results confirm that the hydrogel enriched with grape skin extract not only accelerates wound closure (as shown previously, Figure 4) but also enhances the quality of healing through significantly improved collagen synthesis and matrix deposition. Previous studies have demonstrated that naringin and caffeic acid, major bioactive constituents of our grape skin extract, significantly enhance hydroxyproline levels at the wound site, indicative of increased collagen synthesis. Therefore, it is reasonable to assume that their sustained release from the hydrogel contributed significantly to improved healing by enhancing collagen synthesis and tissue repair as observed in this study [39,40]. 

It is important to emphasize that diabetic wounds are marked by ongoing oxidative stress and chronic inflammation, which disrupt the normal healing cascade by inducing cellular damage, disrupting redox balance, and prolonging tissue repair processes. These pathological factors not only delay wound closure but also contribute to complications such as infection and poor tissue regeneration, making effective management challenging [41,42]. Therefore, the development of novel therapeutic strategies that specifically target and modulate oxidative stress and inflammatory pathways is essential to improve healing outcomes in diabetic wounds. To elucidate the biochemical mechanisms underlying the accelerated wound healing observed with our hydrogel formulation enriched with grape skin extract (HG+GSE), we systematically assessed its effects on both oxidative stress and inflammatory biomarkers in rat skin and blood samples, respectively. 

**Figure 6:**
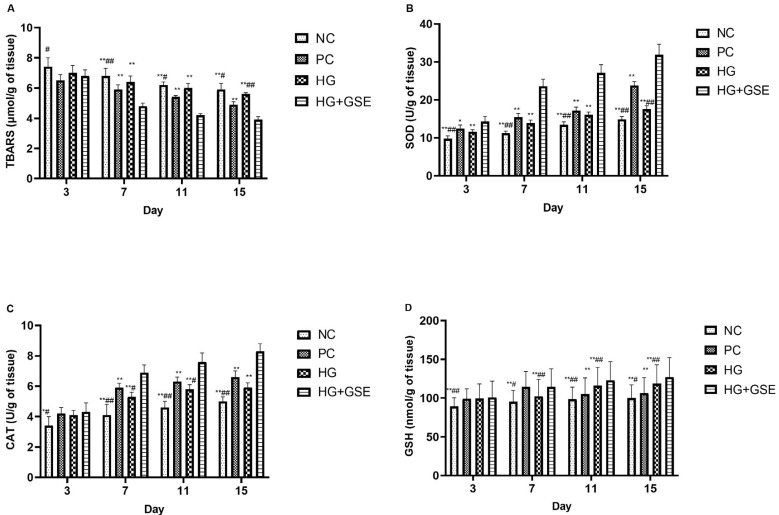
Tissue redox status in rat skin samples. (**A**)—TBARS; (**B**)—SOD; (**C**)—CAT; (**D**)—GSH. Data represent mean ± SD of three independent experiments. NC—nontreated wounds; PC—wounds treated with 1% silver sulfadiazine cream; HG—wounds treated with hydrogel (base hydrogel without extract) and HG+GSE—wounds treated with hydrogel with grape skin extract (GSE); * statistically significant difference compared to HG + GSE at the level *p* < 0.05; ** statistically significant difference compared to HG+GSE at the level *p* < 0.01; ^#^ statistically significant difference compared to PC at the level *p* < 0.05; ^##^ statistically significant difference compared to PC at the level *p* < 0.01.

Therefore, we evaluated the impact of HG+GSE on key oxidative stress markers, such as TBARS, SOD, CAT, and GSH in rat skin tissue, to better understand its role in mitigating oxidative damage and fostering a conducive environment for tissue regeneration. Oxidative stress, characterized by an imbalance between reactive oxygen species production and the antioxidant defense system, is a pivotal factor influencing the progression and resolution of wound healing, especially in chronic conditions such as diabetic wounds [37]. Evaluating oxidative stress parameters allows a deeper understanding of how the hydrogel enriched with GSE modulates the redox balance at the wound site, thereby influencing the healing process. Our data strongly indicates that HG+GSE treatment significantly modulates oxidative stress markers, as illustrated by the data summarized in Figure 6.

Level of TBARS, as an indicator of lipid peroxidation, remained significantly elevated in the NC group throughout the study, suggesting ongoing oxidative damage and impaired wound resolution (Figure 6A). The HG group showed a similar pattern, with only a slight decline over time, indicating limited antioxidant capacity of the hydrogel base. Remarkably, the HG+GSE group demonstrated a nearly twofold decrease in TBARS values, from 6.8 ± 0.4 nmol/g on day 3 to 3.9 ± 0.2 µmol/g tissue on day 15. This reduction was statistically significant compared to NC and HG groups from day 7 onward, confirming the potent antioxidative activity of grape skin extract compounds released from the hydrogel matrix. 

Moreover, SOD activity in the HG+GSE group significantly increased from 14.3 ± 1.4 U/g tissue on day 3 to 31.9 ± 2.8 U/g tissue on day 15, marking over a 2-fold increase compared to the NC group by the end of the study (Figure 6B). This pronounced elevation reflects enhanced catalysis of superoxide radicals into less reactive species, thereby protecting tissues from oxidative damage and fostering a favorable environment for tissue repair [43]. In terms of CAT activity, a progressive increase was also observed in the HG+GSE group, representing a 66% enhancement compared to the NC group, which plateaued at 5.0 ± 0.3 U/g tissue. While the HG and PC groups demonstrated moderate CAT activity increases, their effects were not as pronounced (Figure 6C). Reduced glutathione (GSH), a key non-enzymatic antioxidant, followed a similar trend. The HG+GSE group maintained the highest GSH levels, while the final GSH value at the end of the study in the HG+GSE group was approximately 27% higher than that of the NC group. These results collectively suggest that the grape skin extract-loaded hydrogel significantly enhances both enzymatic and non-enzymatic antioxidant defense mechanisms in the wound tissue. The molecular mechanisms underlying these antioxidant effects can be attributed to the synergistic actions of GSE’s polyphenolic constituents. Caffeic acid activates the nuclear factor erythroid 2-related factor 2/heme oxygenase-1 (Nrf2/HO-1) pathway, a master regulator of cellular antioxidant responses that upregulates the expression of SOD, CAT, and enzymes involved in GSH synthesis [32,33,34]. This pathway activation provides a sustained enhancement of endogenous antioxidant capacity rather than merely supplementing exogenous antioxidants. Naringin complements this effect through dual mechanisms: direct free radical scavenging and activation of the HO-1/Nrf2 pathway, while simultaneously suppressing pro-oxidant pathways by inhibiting NF-κB signaling [32,33]. Epicatechin, with its catechol structure, efficiently neutralizes various reactive oxygen species including superoxide radicals, hydroxyl radicals, and peroxynitrite, thereby reducing the oxidative burden on cellular antioxidant systems. Additionally, naringin’s promotion of neovascularization through VEGF upregulation improves tissue oxygenation, reducing hypoxia-induced ROS generation [29,30], while its activation of MMP-2 and MMP-9 facilitates removal of oxidatively damaged matrix components and their replacement with newly synthesized, intact ECM [28,29,30]. The controlled release of these bioactive compounds from the hydrogel ensures sustained modulation of both pro-oxidant and antioxidant pathways throughout the healing period. This antioxidant reinforcement likely contributes to the improved healing outcomes observed in the HG+GSE group, as corroborated by TBARS measurements and wound closure data, underscoring the extract’s therapeutic potential in managing oxidative stress during tissue repair. 

**Figure 7:**
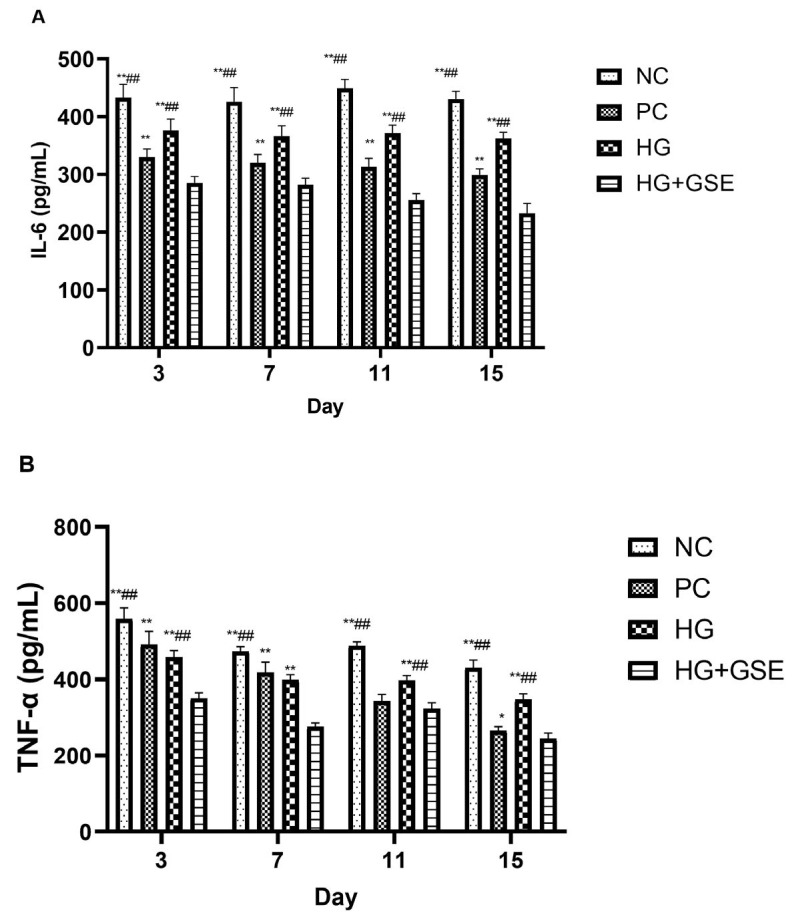
Pro-inflammatory markers in rat blood samples. (**A**)—IL-6; (**B**)—TNF-α. Data represent mean ± SD of two independent experiments. NC—nontreated wounds; PC—wounds treated with 1% silver sulfadiazine cream; HG—wounds treated with hydrogel (base hydrogel without extract) and HG+GSE—wounds treated with hydrogel with grape skin extract (GSE); * statistically significant difference compared to HG+GSE at the level *p* < 0.05; ** statistically significant difference compared to HG + GSE at the level *p* < 0.01; ^##^ statistically significant difference compared to PC at the level *p* < 0.01.

After tissue injury, inflammation initiates the processes of tissue regeneration and repair, thus emphasizing the importance of effective regulation of inflammation for the proper progression of skin damage recovery, healing, and remodeling [41]. Therefore, we sought to gain an understanding of how HG+GSE treatment modulates inflammatory responses throughout the healing process, by determination of serum IL-6 and TNF-α levels on days 3, 7, 11, and 15 post-wounding (Figure 7). In the early phase of wound healing, during the first three days after excision, inflammatory cytokines such as TNF-α and IL-6 play a key role in initiating the immune response, activating leukocytes, and preparing the tissue for the proliferative phase [44,45]. However, excessive or prolonged expression of these mediators can cause damage to the surrounding tissue and disrupt the precise regulation of the regeneration process [46]. In our study, serum concentrations of IL-6 and TNF-α were significantly elevated in the NC and HG groups across all observed time points, reflecting persistent inflammation characteristic of diabetic wounds. In fact, in diabetic rats, chronic hyperglycemia leads to dysregulation of inflammatory responses, manifested by increased and prolonged expression of cytokines like TNF-α and IL-6 [47]. These cytokines, although essential in the initial healing phase, often promote pathological inflammation in the context of diabetes, disrupting the progression to the proliferative phase, which is in accordance with our macroscopic wound closure findings [48]. 

In contrast, the HG+GSE group exhibited a consistent and statistically significant reduction in both IL-6 and TNF-α levels from day 3 onward, indicating effective modulation of the inflammatory response (Figure 7A,B). Notably, by day 15, IL-6 levels in the HG+GSE group dropped nearly two-fold compared to HG (233.3 ± 16.7 pg/mL vs. 362.4 ± 10.6 pg/mL), and almost halved relative to NC group (430.7 ± 13.4 pg/mL). A similar trend was observed for TNF-α, with levels in the HG+GSE group (244.5 ± 14.9 pg/mL) significantly lower than those in both HG group (346.7 ± 15.2 pg/mL) and NC group (430.5 ± 20.1 pg/mL) after 15-day protocol. A reduction in the pro-inflammatory marker levels during this period may reduce the intensity of local inflammation and lower the risk of chronic inflammatory conditions [49,50]. Research suggests that such modulation of the inflammatory response, rather than complete inhibition, can contribute to a faster transition to the proliferative phase and improve overall healing outcomes [51]. 

While the 1% silver sulfadiazine cream also reduced cytokine levels, particularly after day 7, though its effect on TNF-α and IL-6 was less consistent in the early phase. This suggests that 1% silver sulfadiazine may primarily support tissue regeneration rather than directly modulate early inflammatory signaling. In general, topical application of HG+GSE resulted in a statistically significant decrease in TNF-α and IL-6 levels at all monitored time points (days 3, 7, 11, and 15) compared to NC, indicating a potential anti-inflammatory effect of the extract and its ability to modulate the hyperactive inflammatory response characteristic of diabetic wounds [52,53]. The HG and NC failed to produce a similar effect, confirming that the extract is likely the active factor in this modulation. The significant reduction in TNF-α and IL-6 levels observed in the HG+GSE-treated group supports the role of naringin and caffeic acid in modulating inflammatory pathways commonly dysregulated in diabetic conditions. Given that caffeic acid is known to suppress pro-inflammatory mediators such as TNF-α and IL-6 by targeting NF-κB and activating the Nrf2/HO-1 antioxidant defense system, it is likely that its sustained release from the hydrogel contributed to the observed anti-inflammatory effects and improved wound healing outcomes in our diabetic rat model [32,33,34]. Altogether these findings support the hypothesis that moderate and timely reduction in TNF-α and IL-6 not only does not impair healing but can accelerate the transition to subsequent phases and reduce the risk of chronic wounds, which is especially important in models with compromised regenerative capacity, such as diabetic rats [44,49].

#### 3.2.3. Structural Evaluation of Skin Repair via Histological Analysis

Beyond macroscopic assessment of wound contraction and evaluation of oxidative stress and inflammatory markers, histological analysis provides critical insight into the structural and cellular events underlying tissue repair. Histological analysis was particularly important in our study to determine whether the HG+GSE formulation could enhance key histopathological features of healing compared to the NC, PC and HG- treated groups. H/E staining allowed us to assess re-epithelialization and fibroblast activity, while Masson’s Trichrome staining highlighted collagen deposition and matrix organization [54,55]. In the present study, histological evaluation of serial skin sections from the wound area (epidermis and dermis) revealed dynamic and time-dependent tissue responses to injury and treatment. The progression of the reparative process characterized by cicatrization and connective tissue remodeling varied among the experimental groups, with the most rapid and pronounced healing observed in the animals treated with the HG+GSE formulation. Compared to NC, these animals showed accelerated restoration of tissue architecture and more advanced stages of healing at each time point. The HG group showed only modest differences in the intensity and rate of tissue repair compared to the NC and PC groups, indicating that the combination of GSE with the hydrogel matrix offers a more pronounced synergistic effect in promoting structural tissue regeneration.

**Figure 8:**
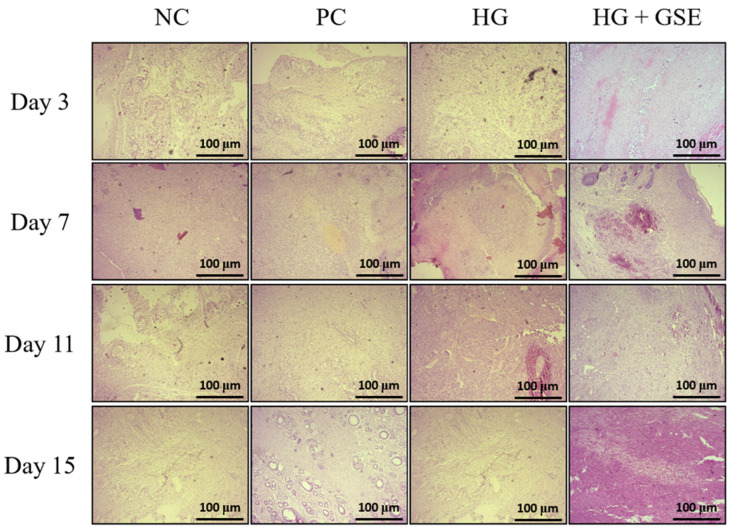
Histological Evaluation of Skin Tissue Using H&E Staining. Magnification 100×; scale bar = 100 µm. NC—nontreated wounds; PC—wounds treated with 1% silver sulfadiazine cream; HG—wounds treated with hydrogel (base hydrogel without extract) and HG+GSE—wounds treated with hydrogel with grape skin extract (GSE).

Additionally, re-epithelialization, defined by the restoration of the continuous stratified squamous epithelium of the epidermis, was completed by day 15 in HG+GSE group. Moreover, earlier signs of epithelial closure and more organized epidermal layers were consistently observed in the HG+GSE group compared to other groups. Changes in the dermis progressed through all stages of fibroplasia from vascular granulation tissue and fibrovascular granulation tissue to maturation and remodeling of mature connective tissue with collagen deposition and transformation of fibroblasts into fibrocytes. Importantly, rats treated with HG+GSE exhibited the greatest tissue maturation and hypocellularity (Figure 8). 

**Figure 9:**
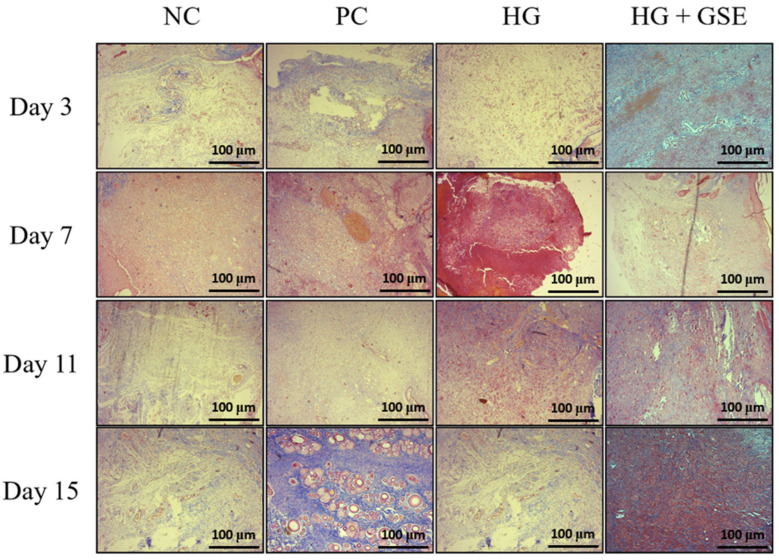
Histological Evaluation of Skin Tissue Using Masson Staining. Magnification 100×; scale bar = 100 µm. NC—nontreated wounds; PC—wounds treated with 1% silver sulfadiazine cream; HG—wounds treated with hydrogel (base hydrogel without extract) and HG+GSE—wounds treated with hydrogel with grape skin extract (GSE).

**Figure 10:**
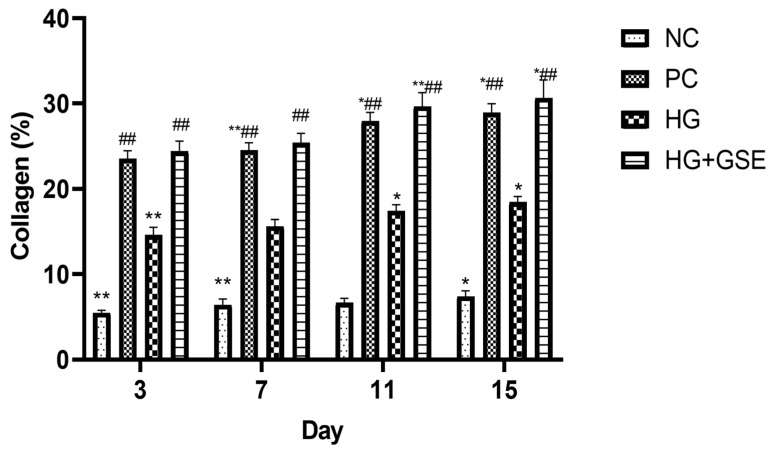
Collagen content in skin tissue. NC—nontreated wounds; PC—wounds treated with 1% silver sulfadiazine cream; HG—wounds treated with hydrogel (base hydrogel without extract) and HG+GSE—wounds treated with hydrogel with grape skin extract (GSE); * statistically significant difference compared to HG+GSE at the level *p* < 0.05; ** statistically significant difference compared to HG + GSE at the level *p* < 0.01; ^##^ statistically significant difference compared to PC at the level *p* < 0.01.

Additionally, the amount of deposited collagen was highest in the HG+GSE treated rats, while in contrast, animals treated with 1% silver sulfadiazine cream and the hydrogel base exhibited fewer collagen fibers and a predominance of hypercellular connective tissue characterized by abundant active fibroblasts. This implies that tissue remodeling is less advanced in PC and HG groups than in those receiving the HG+GSE treatment. Meanwhile, in the NC, the wound healing process progressed at a slower pace, with noticeably reduced collagen deposition and delayed maturation of fibroblasts into fibrocytes, as confirmed by Masson’s Trichrome staining (Figure 9). The HG+GSE group consistently showed significantly higher collagen content, increasing from 24.41 ± 1.2% on day 3 to 30.68 ± 2.2% on day 15, compared to the negative control (5.46 ± 0.3–7.4 ± 0.67%) and HG alone (14.6 ± 0.9–18.43 ± 0.68%), and reaching levels similar to the positive control (23.57 ± 0.91–28.97 ± 1.01%), indicating that the combined formulation effectively promotes collagen deposition (Figure 10).These findings highlight the enhanced ability of the GSE-containing hydrogel to promote extracellular matrix synthesis and accelerate tissue maturation during wound repair (Figure 9 and Figure 10).

The release of naringin from our hydrogel formulation likely played a significant role in the observed improvements in tissue structure. It has been shown that naringin-based formulations enhanced tissue restoration during wound healing. This effect aligns with naringin’s well-documented ability to promote histological repair by upregulating angiogenic growth factors such as VEGF-A, VEGF-B, VEGF-C, and their receptor VEGF-R3. Literature data also indicates that this upregulation stimulates matrix metalloproteinases 2 and 9, which are essential for extracellular matrix remodeling and tissue regeneration [28,29,30]. Consequently, naringin’s release from the hydrogel may have facilitated greater collagen deposition, faster fibroblast maturation, and overall enhanced tissue architecture as evidenced by our histological findings. Additionally, caffeic acid may also play a notable role in tissue structure improvement by enhancing epithelial restoration, collagen organization, reduced inflammation, and neovascularization, as it was shown in a previous study [40].

In addition to promoting wound healing, the multifunctional HG+GSE hydrogel demonstrated antimicrobial activity against clinically relevant pathogens, including *Escherichia coli* in our previous study [13]. This suggests that the formulation could be particularly suitable for the management of early diabetic wounds, potentially preventing infection and the progression to chronic non-healing wounds.

The histological improvements observed in the HG+GSE group can be mechanistically explained by the complementary actions of the extract’s bioactive constituents. Naringin’s release from the hydrogel formulation likely played a pivotal role in tissue structural restoration through its well-documented ability to upregulate angiogenic growth factors such as VEGF-A, VEGF-B, VEGF-C, and their receptor VEGF-R3 [28,29,30]. This upregulation stimulates neovascularization, ensuring adequate oxygen and nutrient supply to support cellular metabolic demands during the proliferative phase. Simultaneously, naringin’s induction of matrix metalloproteinases 2 and 9 facilitates extracellular matrix remodeling, allowing for organized collagen deposition rather than the disorganized scarring typical of diabetic wounds [28,29,30]. Consequently, naringin’s release from the hydrogel may have facilitated greater collagen deposition, faster fibroblast maturation into fibrocytes, and overall enhanced tissue architecture as evidenced by our Masson’s trichrome staining. Additionally, caffeic acid contributed substantially to tissue structure improvement through multiple mechanisms: enhancing epithelial restoration by promoting keratinocyte migration and proliferation, facilitating organized collagen synthesis through modulation of transforming growth factor-β (TGF-β) signaling, reducing inflammation-mediated tissue damage via NF-κB inhibition, and promoting neovascularization through endothelial cell activation [40]. Epicatechin’s antioxidant activity further supported these processes by protecting newly synthesized collagen and other ECM components from oxidative degradation, ensuring the structural integrity of the regenerated tissue. The sustained and controlled release of these compounds from our hydrogel formulation ensured continuous therapeutic effects throughout all phases of healing, from the inflammatory phase through proliferation and into remodeling, ultimately resulting in the superior histological outcomes observed in the HG+GSE group.

Although HG+GSE demonstrated superior wound healing compared to 1% silver sulfadiazine, further comparisons with additional commercial products are planned. This study was limited to animal models, so clinical trials and cost-effectiveness analyses are necessary to comprehensively evaluate safety, efficacy, and economic impact in humans. However, the use of natural hydrogels enriched with multifunctional bioactive compounds aligns with current trends in sustainable and personalized medicine, offering promising prospects for future development.

## 4. Conclusions

This study demonstrates that the GSE-enriched hydrogel (HG+GSE) represents a promising, biocompatible dressing for diabetic wound healing. Its application significantly accelerated wound closure, enhanced collagen deposition, and promoted tissue remodeling, outperforming both base hydrogel and conventional silver sulfadiazine treatment. Importantly, the hydrogel reduced oxidative stress and pro-inflammatory cytokines while enhancing key antioxidant defenses, confirming the multifunctional therapeutic role of grape skin polyphenols. 

Unlike previous studies that mainly investigated grape seed or pomace extracts for their general antioxidant or antimicrobial benefits, this work specifically evaluated the in vivo wound-healing efficacy of extracts from Serbian grape skins incorporated into biodegradable alginate–gelatin hydrogels. This study thus provides preclinical evidence of efficacy in a diabetic wound model, supported by macroscopic, biochemical, and histological analyses. The mechanistic basis for these therapeutic effects lies in the complementary actions of GSE’s major constituents: naringin’s promotion of angiogenesis and matrix remodeling through VEGF and MMP upregulation, caffeic acid’s dual suppression of inflammatory pathways (NF-κB) and activation of antioxidant defenses (Nrf2/HO-1), and epicatechin’s potent ROS scavenging activity. This multi-targeted approach addresses the complex pathophysiology of diabetic wounds more comprehensively than conventional single-agent therapies. Findings of the present study not only validate the efficacy of HG+GSE in a diabetic model but also advocate for the valorization of grape industry byproducts in biomedical applications. Future studies should focus on the potential clinical translation of this eco-friendly, phytochemical-loaded hydrogel system.

## Data Availability

The data presented in this study are available in this article.
